# iPSC model of *CHRFAM7A* effect on α7 nicotinic acetylcholine receptor function in the human context

**DOI:** 10.1038/s41398-019-0375-z

**Published:** 2019-02-01

**Authors:** Ivanna Ihnatovych, Tapan K. Nayak, Aya Ouf, Norbert Sule, Barbara Birkaya, Lee Chaves, Anthony Auerbach, Kinga Szigeti

**Affiliations:** 1 Department of Neurology, State University of New York at Buffalo, Buffalo, NY USA; 2 Department of Physiology and Biophysics, State University of New York at Buffalo, Buffalo, NY USA; 3 Kusuma School of Biological Sciences, IIT Delhi, Hauz Khas, New Delhi, 110016 India; 4 Department of Pathology, Roswell Park Cancer Institute, Buffalo, NY USA; 5 Division of Nephrology, Department of Medicine, State University of New York at Buffalo, Buffalo, NY USA

## Abstract

The α7 nicotinic acetylcholine receptor (α7nAChR) has been a promising target for diseases affecting cognition and higher cortical functions; however, the effect observed in animal models failed to translate into human clinical trials identifying a translational gap. *CHRFAM7A* is a human-specific fusion gene with properties that enable incorporation into the α7nAChR and, being human specific, *CHRFAM7A* effect was not accounted for in preclinical studies. We hypothesized that *CHRFAM7A* may account for this translational gap and understanding its function may offer novel insights when exploring α7nAChR as a drug target. *CHRFAM7A* is present in different copy number variations (CNV) in the human genome with high frequency. To study the functional consequences of the presence of the *CHRFAM7A*, two induced pluripotent stem cell (iPSC) lines (0 copy and 1 copy direct) were developed. The 0 copy line was rescued with *CHRFAM7A* transfection to control for genetic heterogeneity. As readouts for genotype–phenotype correlation, α7nAChR synaptic transmission and amyloid beta 1–42 (Aβ_1–42_) uptake were tested. Synaptic transmission in the presence of *CHRFAM7A* demonstrated that PNU-modulated desensitization of α7nAChR currents increased as a function of *CHRFAM7A* dosage. *CHRFAM7A* mitigated the dose response of Aβ_1–42_ uptake suggesting a protective effect beyond physiological concentrations. Furthermore, in the presence of CHRFAM7A Aβ_1–42_ uptake activated neuronal interleukin 1β (IL-1β) and tumor necrosis factor α (TNF-α) without activating the canonical inflammasome pathway. Lead optimization may identify more potent molecules when the screen has a model harboring *CHRFAM7A*. Incorporating pharmacogenetics into clinical trials may enhance signals in efficacy measures.

## Introduction

The α7 nicotinic acetylcholine receptor (α7nAChR) is a ligand-gated ion channel implicated in cognition and neuropsychiatric disorders, including schizophrenia^1–3^, Alzheimer’s disease (AD)^4,5^ attention deficit hyperactivity disorder^6^, addiction^7^, pain^8,9^, and Parkinson disease^10^. Agonists and positive allosteric modulators (PAMs) of α7nAChR are being tested in clinical trials for central nervous system indications^11^, and a translational gap emerged^12^. While animal studies consistently demonstrate a cognitive benefit, this effect was not apparent in human trials^13,14^.

The α7nAChR is a homopentamer characterized by unique functional properties, including fast activation and desensitization by agonists, high Ca^2+^ permeability, and selective inhibition by α-bungarotoxin (α-BGT) and methyllycaconitine (MLA)^12,15^. It is expressed in brain regions underlying cognition and memory^16^, including the basal forebrain (nucleus basalis), hippocampus, neocortex, and amygdala^17,18^, α7nAChRs are expressed in neurons, microglia and astrocytes^19^. The α7nAChRs are regulators of the cholinergic anti-inflammatory pathway^20,21^, and acetylcholine (ACh) produces a dose-dependent inhibition of interleukin 6 (IL-6), IL-1β, and tumor necrosis factor α (TNF-α) in human macrophages^22^. In neuronal cell culture, Aβ_1–42_ binds with high affinity to the α7nAChRs and the receptor facilitates internalization of Aβ_1–42_ through endocytosis^23^.

One of the unique features of the α7nAChR in the human context is the presence of the fusion gene, *CHRFAM7A*^24,25^, The ancestral allele lacking the fusion gene is present in about 1% of the population, while 99% of the human population harbors the fusion gene. Further complexity is added as the orientation of *CHRFAM7A* can be direct or inverted, and the gene itself can be present in 1, 2, or even 3 copies, allowing for homozygous and heterozygous combinations. The predicted proteins of the direct and inverted alleles differ due to a 2 bp deletion in the inverted sequence causing a frameshift during protein translation^26^.

*CHRFAM7A* harbors exons 5–10 of *CHRNA7* (transmembrane and intracellular domain) and 5 new exons of the *FAM7* sequence (extracellular domain) corresponding to a part-functional *CHRNA7*. Two independent copy number variation (CNV) genome-wide association studies (GWAS) reported an association between *CHRFAM7A* dosage and AD; lower copy number and lower expression levels of the fusion gene is associated with AD^27–29^, In contrast, in schizophrenia and bipolar disorder, upregulation of *CHRFAM7A* was observed in the brain^3^ and association studies suggest a correlation with the inverted orientation (2 bp deletion)^26^. Despite its widespread implication in neuropsychiatric diseases, functional studies are sparse. In *CHRFAM7A*-transfected Xenopus oocytes, *CHRFAM7A* is a stoichiometric dominant-negative regulator of α7nAChR^30,31^.

Owing to the high frequency and complexity of the *CHRFAM7A* CNV in the human population, understanding its functional impact is imperative for interpreting α7nAChR targeting clinical trials. To study the functional consequences of the presence of the *CHRFAM7A* gene product on α7nAChR, we developed two induced pluripotent stem cell (iPSC) lines from skin biopsies of subjects affected by AD. UB068 has two ancestral haplotypes, thus it is lacking the *CHRFAM7A* gene (0 copy). UB052 has the *CHRFAM7A* direct orientation haplotype on one allele and the ancestral haplotype on the other allele (1 copy). iPSCs differentiated into relevant cell types, in our case medial ganglionic eminence (MGE) progenitors and neurons, model the effect of *CHRFAM7A* on α7nAChR function in the human context. As readouts for genotype–phenotype correlation, α7nAChR synaptic transmission and Aβ_1–42_ uptake were tested.

## Materials and methods

### Ethical statement, skin biopsy, and genotyping

The Institutional Review Board approved the study. The informed consents were obtained from the donors. Subjects requiring legally authorized representatives were excluded from the study.

### iPSC generation and cell culture

iPSC lines (UB068—0 copy and UB052—1 copy direct) were generated from human skin biopsies in WNYSTEM (University at Buffalo) by episomal transformation and propagated in standard media (Supplementary data, Methods). iPSCs characterization according the industry standards included morphological assessment and live staining with the TRA-1–60 Alexa Fluor 488 Conjugate Kit (Life Technologies). Gene and protein expression for pluripotency/self-renewal and the three germ layer markers at gene and protein levels was assessed by reverse transcription–quantitative polymerase chain reaction (RT–qPCR) and immunocytochemistry (ICC) (Supplementary data, Methods; The primers (*IDT*) are listed in Supplementary data, Table 1, The primary antibodies are listed in Supplementary data, Table 2). Array comparative genome hybridization (aCGH) was performed between the iPSC colony and the original blood DNA sample from the same individual according to the manufacturer’s protocol. The ADM2 algorithm with a threshold of 6 was used to detect de novo events.

Pluripotency of the iPSC was confirmed by the TaqMan hPSC Scorecard Assay (Life Technologies) according to the manufacturer’s protocol^32,33^.

### Neuronal differentiation and transfection

Neuronal differentiation of iPSC toward MGE progenitors and neurons was carried out using the protocol based on Liu et al.^34^ with modifications (Supplementary data, Methods). MGE progenitors were transfected with either pcDNA3.1-CHRFAM7A-mCherry (a gift from Henry Lester (Addgene plasmid # 62635)^35^) or with pcDNA3.3-mCherry (a gift from Derrick Rossi (Addgene plasmid # 26823)^36^ constructs according to Ma et al.^37^.

### Total cell lysate preparation and immunoblotting

Total cell lysates from MGE progenitors were prepared using RIPA buffer (Cell Signaling Technologies) according to the manufacturer’s protocol. Twenty-five μg of total protein was separated on 4–20% sodium dodecyl sulfate–polyacrylamide gel electrophoresis (Bio-Rad), transferred onto polyvinylidene difluoride membrane (Bio-Rad), and incubated overnight at 4 °C with primary antibodies (Supplementary data, Table 2). Specific immunoreactive bands were detected using ChemiDoc XRS + Imaging Systems (Bio-Rad).

### α-BGT staining and confocal microscopy

Live α-BGT staining of neurons was carried out as described previously^38^. Briefly, neurons grown on 8-well glass chambers were pre-incubated for 10 min with 1 mM Nicotine followed by incubation for 30 min with 2 μM with α-BGT. Confocal images were captured by using LSM510 Meta microscope (×40 objective). Images were acquired using the ZEN black software (Zeiss).

### HEK 293 cell culture and transfection

Human embryonic kidney (HEK) 293 cells were maintained in Dulbecco’s Minimal Essential Medium supplemented with 10% fetal bovine serum and 1% penicillin–streptomycin, pH 7.4. Human α7nAChRs and *CHRFAM7A* were expressed in HEK 293 cells by transient transfection (CaPO_4_ precipitation method) of these cDNA in ratios of 4:1 or 1:4. To aid surface expression of α7, we co-transfected intracellular chaperones Ric-3^39^ and NACHO^40^ in 1:1:1 ratio in all experiments.

### Electrophysiology

Whole-cell and single-channel currents were recorded in the cell-attached patch configuration as described previously^41^ (Supplementary data, Methods). Kinetic analyses of single-channel currents were performed by using QuB^42^. Single-channel currents were idealized by segmental *k*-means algorithm. *n*Po was estimated by dividing the cumulative open probability by the number of channels in the patch (maximum number of overlaps of open current levels in the data) as follows:

$${\mathrm{Po} = \frac{{\mathop {\sum }\nolimits^ n{\mathrm{Po}}}}{n}}$$

### Amyloid beta uptake and quantification

Amyloid beta uptake was performed using fluorescently labeled Aβ_1–42_ (AnaSpec) as described previously^43^. Briefly, MGE progenitors grown on glass coverslips were treated with various concentrations of Fluorescin-Aβ_1–42_ (1, 10, 25, 50, 100, and 250 nM) for 18 h. Live images were taken using EVOS (Life Technologies) microscope (×40 objective). Confocal images were taken with the LSM510 Meta microscope (×40 objective). Amyloid beta uptake was quantified by using ImageJ (imagej.nih.gov) or by Flow cytometry using LSRII-Fortessa with FACS DIVA (BD Biosciences). The flow cytometry data were analyzed using the FlowJo software (https://www.flowjo.com/). Data are presented as the average of triplicates ± standard deviation (SD).

### IL-1β enzyme-linked immunosorbent assay (ELISA)

Following transfection and amyloid beta uptake, total cell lysates were collected and stored at −80 °C. Concentration of IL-1β in the cell lysates was estimated using a human-specific high-sensitivity IL-1β ELISA Kit (Thermo Fisher) according to the manufacturer’s protocol.

### Statistical analysis

Values are expressed as means ± SD or ±SEM, as indicated in figure legends. Statistical significance was determined by an unpaired Student’s *t*-test (two-tailed). *P* values <0.05 were deemed statistically significant.

## Results

### iPSC characterization

**Fig. 1:**
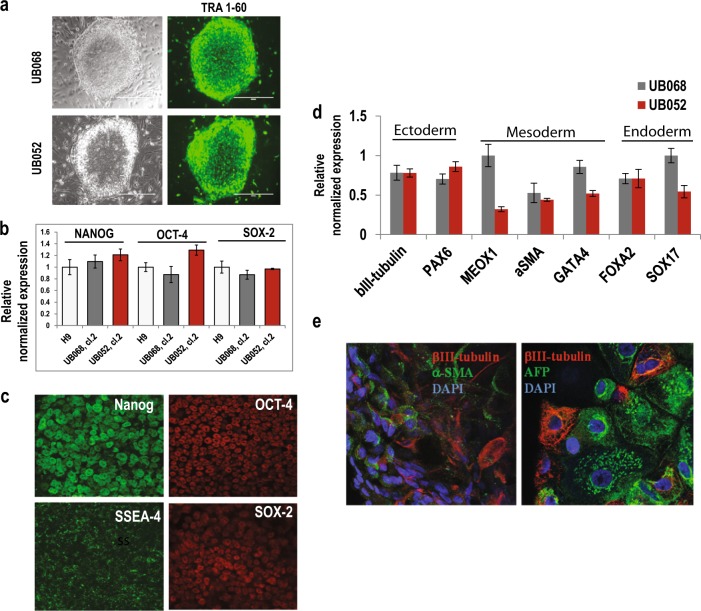
Characterization of human induced pluripotent stem cell (iPSC) lines. **a** Live images of the UB068 (0 copy) and UB052 (1 copy of *CHRFAM7A*) colonies stained with TRA1–60 antibody. **b** Reverse transcription–quantitative polymerase chain reaction analysis of self-renewal/pluripotency gene expression in iPSC lines in comparison with H9 cell line. **c** Confocal images of the iPSCs probed for Nanog, Oct4, Sox2, and SSEA-4 proteins. Gene (**d**) and protein (**e**) expression of the three germ layer markers at day 15 of non-directed differentiation in UB068 and UB052 lines

Stem cell characteristics of new iPSC lines UB068 (0 copy) and UB052 (1 copy) were confirmed by morphology; TRA1–60 live cell staining (Fig. 1a); detecting expression of pluripotency/self-renewal markers (*NANOG*, *OCT-4*, *and SOX2*) (Fig. 1b); and by ICC for Nanog, Oct-4, SOX2 and SSEA-4 (Fig. 1c). aCGH between the iPSC colony and the original blood DNA excluded de novo chromosomal aberrations and CNVs (Fig. 1a, Supplementary data). TaqMan assay specific to the breakpoint sequence confirmed *CHRFAM7A* CNV dosage (Fig. 1b, Supplementary data).

Pluripotency of iPSC lines was confirmed by embryoid body (EB)-based non-directed differentiation (Fig. 1c, Supplementary data), gene expression (Fig. 1d), and ICC with anti-βIII-tubulin (ectoderm), anti-α-SMA (mesoderm), and anti-α-fetoprotein (AFP, endoderm) antibodies (Fig. 1e) and by the TaqMan hPSC Scorecard Assay (ThermoFisher) (Fig. 1d, Supplementary data).

### Neuronal differentiation

**Fig. 2:**
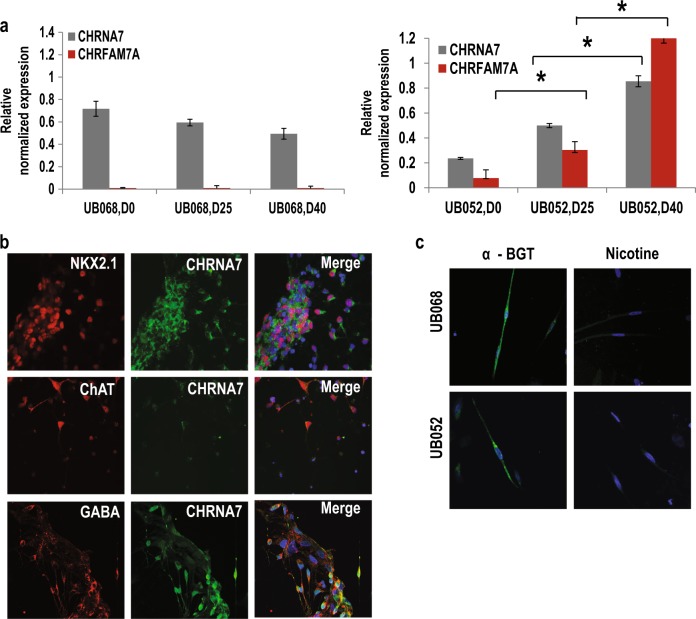
Expression of CHRNA7 and CHRFAM7A during neuronal differentiation of induced pluripotent stem cells (iPSCs). **a** Reverse transcription–quantitative polymerase chain reaction analysis of *CHRFAM7A* and *CHRNA* expression throughout the course of neural differentiation: D0 (pluripotent iPSC), D25 (MGE progenitors), and D40 (neurons). Primers amplify the unique breakpoint sequence and distinguish between the CHRFAM7A and CHRNA7 mRNA. Data are presented as mean ± SEM. *P < 0.05 difference between *CHRFAM7A* expression at D0 and D25, and D40. **b** Confocal images demonstrating expression of CHRNA7/CHRFAM7A in MGE progenitors (NKX 2.1), GABA interneurons (GABA), and BFCN (choline acetyltransferase). Note that CHRNA7 and CHRMFAM7A cannot be distinguished with antibodies. **c** α7nAChR (α7 nicotinic acetylcholine receptor)-specific live staining of UB068- and UB052-derived neurons with α-bungarotoxin (α-BGT). Preincubation with nicotine prevents fluorescein isothiocyanate–α-BGT binding

Based on the TaqMan hPSC Scorecard Assay clones with the highest preference for neuronal differentiation (UB068, clone 2 and UB052, clone 2) were chosen for further experiments. UB068 (0 copy) and UB052 (1 copy) clones were differentiated into MGE progenitors^34^ and MGE-derived BFCNs and GABA interneurons. Expression levels of *NKX2.1*, *LHX6*, and *LHX8* (MGE markers), *FOXG1* (forebrain marker), *PAX6 (*dorsal forebrain marker*) SOX2* and *MAP-2* (pan neuronal markers), and *ChAT*, *GAD*, *TH*, and *HB9* (BFCN, GABA, dopaminergic, and motor neuron markers, respectively) were quantified during neuronal differentiation (Fig. 2a, Supplementary data). MGE progenitors demonstrated high level of *FOXG1*, *NKX2.1*, *LHX6*, and *LHX8* expression detected at D25 and MGE-derived GABA and BFCN showed high level of *GAD* and *ChAT* expression and very low level of *TH* and *HB9* expression at D40. ICC confirmed GABA and/or choline acetyltransferase (ChAT) expression in neurons (Fig. 2b, Supplementary data). Spontaneous action current activity and voltage-gated Na+ and K+ currents recorded from UB068- and UB052-derived neuronal cultures confirmed functional neurons (Fig. 2c, Supplementary data). Single-channel patch-clamp analysis confirmed α7-specific currents in neurons (Fig. 2d, Supplementary data).

### *CHRFAM7A* gene expression during neuronal differentiation

*CHRNA7* and *CHRFAM7A* breakpoint (unique sequence) specific primers demonstrated expression of *CHRNA7* (in UB068 and UB052) and *CHRFAM7A* (in UB052) during neuronal differentiation. The results of RT–qPCR revealed a two-fold increase in *CHRNA7* expression at D40 compared to D0 in UB052 cells (*P* = 0.045), while no changes in *CHRNA7* expression were detected in UB068 cells (Fig. 2a, left and right panel). *CHRFAM7A* expression level was upregulated six times during the process of neuronal differentiation in UB052 (*P* = 0.027) [Fig. 2a, right panel]. ICC demonstrated co-expression of CHRNA7 and NKX2.1 in MGE progenitors (Fig. 2b) and MGE-derived neurons (both BFCN and GABA interneurons) generated from UB068 and UB052 lines (Fig. 2b). Live staining with α-BGT demonstrated the presence of functional α7nAChR in both UB068- and UB052-derived neurons (Fig. 2c).

### *CHRFAM7A* is a modulator of the α7nAChR

The effect of *CHRFAM7A* on α7nAChRs can be attributed to altered biophysical properties and/or surface expression of the channels. To investigate the possible changes in the kinetic properties of α7nAChRs, single-channel cell-attached (mechanism of action) and whole-cell patch-clamp (surface expression) recordings on UB068 and UB052 neurons were performed.

**Fig. 3:**
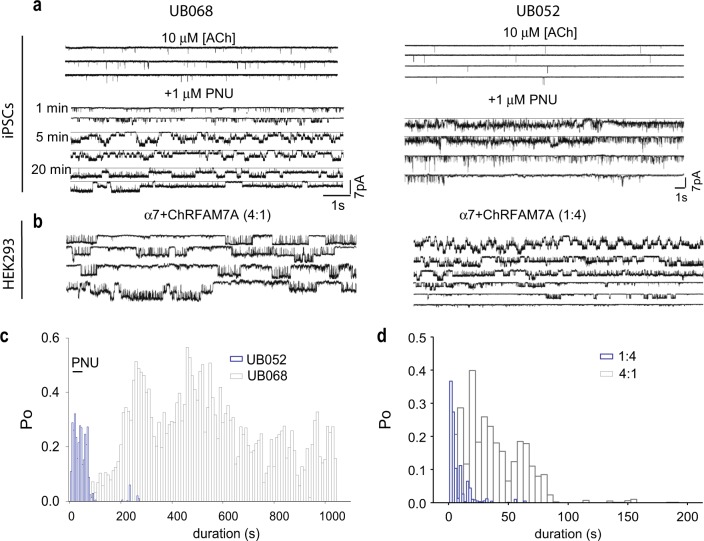
Effect of CHRFAM7A on electrophysiological properties of α7 nicotinic acetylcholine receptors (α7nAChRs). **a** Single-channel current recordings in cell-attached mode from neurons derived from UB068 (left panel) and UB052 (right panel) cell lines in the presence of TTX and TAE. Note the strong potentiation of α7 nAChRs currents in the presence of PNU. **b** Effect of CHRFAM7A on α7nAChR stoichiometry and functional properties in HEK 293 cells transfected with α7nAChR and CHRFAM7A cDNA in 4:1 (left panel) and 1:4 proportions (right panel); PNU differentially modulates currents from HEK 293 cells expressing α7nAChR and CHRFAM7A in 4:1 and 1:4 ratio. **c** Comparative *n*Po analyses of currents from both induced pluripotent stem cell lines showing difference in kinetic properties between the α7nAChR types expressed in them. **d** *n*Po analyses of both currents in HEK 293 cells transfected with the target genes

Functional expression of neuronal α7nAChRs was studied by recording cell-attached single-channel currents from UB068 (0 copy) and UB052 (1 copy) cell lines in the presence of TTX and TAE (Fig. 3a, left and right panels). α7nAChR expression in both cell types albeit lower expression in case of UB052 was observed. α7nAChRs currents were pharmacologically characterized by utilizing the PAM of α7nAChR, PNU 120596 (PNU). The effects of PNU on single α7nAChR currents from UB068 and UB052 cells are depicted in Fig. 3a, left and right panels. PNU in UB068 cells progressively increased channel open probability of single α7nAChRs in a time-dependent manner^44,45^, On the other hand, in UB052-derived neurons, PNU-modulated currents desensitized/ran down faster than in neurons generated from UB068. Comparative *n*Po analyses of currents from both cell types showed the differences in CHRFAM7A effects on the channel kinetic properties (Fig. 3c).

To probe the effect of *CHRFAM7A* on α7nAChR stoichiometry and functional properties, we heterologously expressed them by transfecting *CHRNA7* and *CHRFAM7A* cDNA in HEK 293 cells in 4:1 and 1:4 proportions. The dosage of cDNA was expected to determine the stoichiometry of the α7nAChR expressed in HEK 293 cells. PNU modulated currents from HEK 293 cells expressing α7nAChR and CHRFAM7A both in 4:1 and 1:4 ratios (Fig. 3b, left and right panels respectively). Qualitatively, the single-channel current clusters appeared similar in both cases. However, *n*Po analyses of both currents suggest that desensitization of PNU-modulated α7nAChR currents increased as a function of *CHRFAM7A* dosage (Fig. 3d), consistent with the results contrasting UB068 and UB052.

### Amyloid beta uptake via the α7nAChR is mitigated by *CHRFAM7A*

**Fig. 4:**
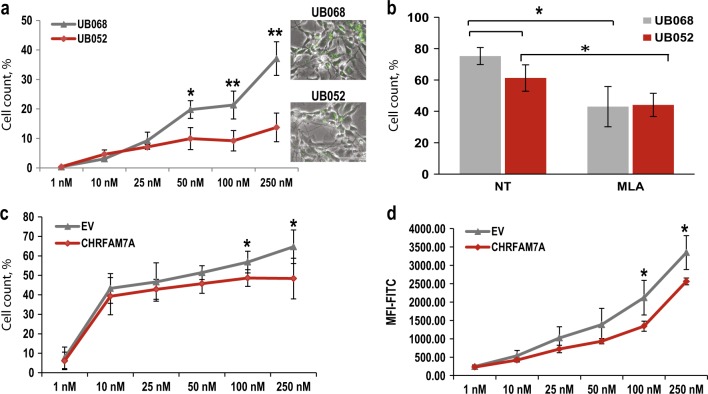
CHRFAM7A modulates amyloid beta uptake via the α7 nicotinic acetylcholine receptor. **a** Difference in concentration-dependent Aβ_1–42_ uptake by MGE progenitors derived from UB068 and UB052 lines. Data are presented as mean ± SD. *P < 0.05 **P < 0.001—difference between Aβ_1–42_ uptake in UB052 line compared to UB068 at each given Aβ_1–42_ concentration. Inset: representative EVOS images of Aβ_1–42_ (100 nM) uptake in both cell lines. **b** Pretreatment with α7-selective antagonist methyllycaconitine (MLA; 10 μM) leads to a significant decrease in A_β1–42_ (100 nM) uptake in both cell lines. Data are presented as mean ± SD. *P < 0.05—difference between Aβ_1–42_ uptake in MGE progenitors with and without treatment (NT) with MLA. Transfection of UB068 (0 copy) with *CHRFAM7A* causes a decrease in Aβ_1–42_ uptake in a concentration-dependent manner compared to transfection with empty vector (EV) analyzed by cell counts (**c**) and as mean fluorescent intensity by flow cytometry (**d**). Data are presented as mean ± SD. *P < 0.05—difference between Aβ_1–42_ uptake in *CHRFAM7A*-transfected cells compared to EV-transfected cells at each given Aβ_1–42_ concentration

Aβ_1–42_ uptake in MGE progenitors derived from UB068 (0 copy), and UB052 (1 copy) lines was quantified by cell counts (live EVOS and confocal images, ImageJ) and flow cytometry. The results demonstrated higher Fluorescin-Aβ_1–42_ uptake in UB068 in contrast to UB052 (Fig. 4a). Furthermore, while the uptake was dose dependent (correlating with increasing concentration of Fluorescin-Aβ_1–42_ from 1 to 250 nM) in UB068, it was constant between concentrations of 25–250 nM in UB052 (Fig. 4a). This suggests that *CHRFAM7A* may be functioning as a regulator of Aβ_1–42_ uptake beyond physiological concentrations.

Pharmacological modulation of the α7nAChR affected Aβ_1–42_ uptake suggesting an α7AChR-dependent mechanism. Pretreatment of MGE progenitors with the α7-selective antagonist MLA resulted in a significant decrease in Aβ_1–42_ uptake in neuronal progenitor cells (NPCs) derived from UB068 (from 74 to 43%; *P* = 0.009) and from UB052 (from 58 to 44%; *P* = 0.037) (Fig. 4b).

To exclude that the observed effect is caused by the different genetic background in the two lines, UB068-derived NPCs were transfected with pcDNA3.1-*CHRFAM7A*-mCherry cDNA (controls: pcDNA3.3-mCherry empty vector (EV)). Efficiency of transfection was estimated by counting mCherry-positive cells and confirmed by qPCR with specific primers (Fig. 3, Supplementary data). After 24 h of transfection, the cells were treated for 18 h with the concentration gradient of Fluorescin-Aβ_1–42_ (1–250 nM)_._ Aβ_1–42_ uptake demonstrated that the presence of *CHRFAM7A* leads to a decrease in Aβ_1–42_ uptake (Fig. 4c). Furthermore, the regulatory effect of CHRFAM7A on Aβ_1–42_ uptake observed in UB052-derived NPCs between the concentrations from 25 to 250 nM was replicated. In contrast, transfection of the NPC with EV pcDNA3.3-mCherry resulted in a dose–response Aβ_1–42_ uptake similar to non-transfected UB068. Orthogonal quantification by flow cytometry corroborated these results (Fig. 4d and Fig. 4, Supplementary data).

### Activation of inflammatory pathways by Aβ_1–42_ through the α7nAChR is mitigated by *CHRFAM7A*

**Fig. 5:**
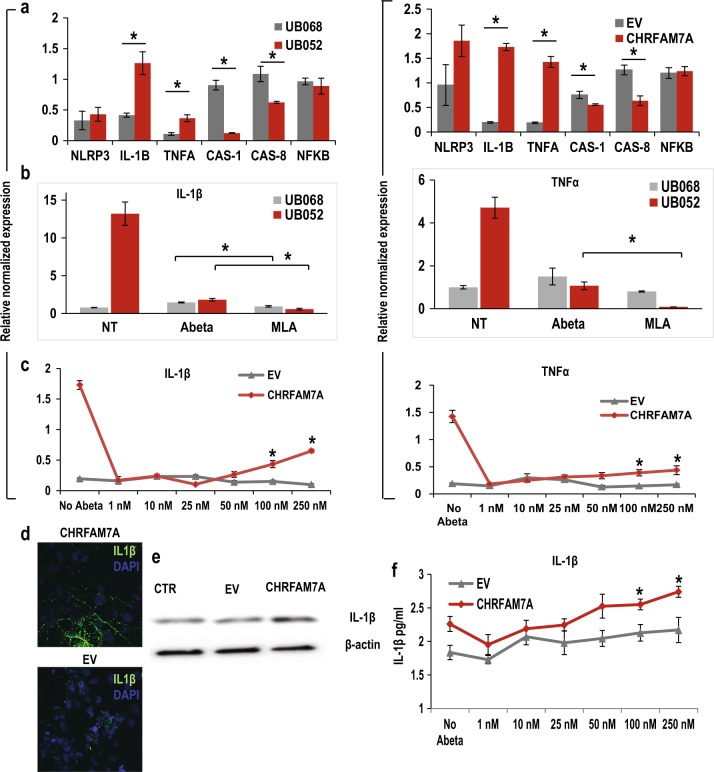
Activation of inflammatory pathways by Aβ_1–42_ through the α7 nicotinic acetylcholine receptor is mitigated by *CHRFAM7A* **a** Comparison of inflammasome-related gene expression profiles in neuronal progenitor cells (NPCs) generated from UB068 and UB052 (left panel) and in UB068 transfected with *CHRFAM7A* and/or empty vector (*EV)* (right panel). Data are presented as mean ± SEM. * - P < 0.05 - difference in gene expression levels in UB052 cells compared to UB068 and in *CHRFAM7A*-transfected cells compared to EV-transfected cells. **b** Pretreatment with α7-selective antagonist methyllycaconitine (MLA) decreases Aβ_1–42_-induced *IL-1β* (left panel) and tumor necrosis factor α (*TNF-α*; right panel) expression in UB068 and UB052 lines. 100 nM Aβ_1–42_ was used for treatment. Data are presented as mean ± SEM. *P < 0.05—difference in Aβ_1–42_-induced gene expression levels between MGE progenitors with and without treatment with MLA in both cell lines. **c** Expression of *IL-1β* (left panel) and *TNF-α* (right panel) correlates with Aβ_1–42_ uptake in concentration-dependent manner in the NPCs transfected with *CHRFAM7A*. Data are presented as mean ± SEM. *P < 0.05—difference in *IL-1β* and/or *TNF-α* expression in *CHRFAM7A*-transfected cells compared to EV-transfected cells at each given Aβ_1–42_ concentration. **d** Representative confocal images and **e** immunoblot analysis of total cell lysates showing an increase in IL-1β expression in the cells transfected with *CHRFAM7A*. **f** In MGE progenitors transfected with *CHRFAM7A*, Fluorescin-Aβ_1–42_ uptake induces a concentration-dependent increase in IL-1β secretion as detected by ELISA. Data are presented as mean ± SD. *P < 0.05—difference between IL-1 β concentration in *CHRFAM7A*-transfected cells compared to EV-transfected cells at each given Aβ_1–42_ concentration

α7nAChRs are regulators of the cholinergic anti-inflammatory pathway and ACh produces a dose-dependent inhibition of IL-6, IL-1β, and TNF-α in macrophages. Therefore, we examined whether this mechanism plays a role in MGE progenitors expressing α7AChRs and whether it is modulated by the presence of CHRFAM7A. We hypothesized that the presence of CHRFAM7A affects the inflammatory pathways activated by *Aβ*_***1–42***_ uptake. We found that expression levels *of IL-1B* and *TNFA* are increased in MGE progenitors expressing *CHRFAM7A* (UB052 and UB068 transfected with *CHRFAM7A*; Fig. 5a—left and right panels), whereas *CAS-1* and *CAS-8* expression are higher in MGE progenitors lacking the fusion gene (UB068 and transfection with EV) suggesting that IL-1β activation in the presence of *CHRFAM7A* is not dependent on the canonical inflammasome pathway. *ΝFΚΒ* is not affected by the presence of *CHRFAM7A* (Fig. 5a—left and right panels). Pretreatment of NPCs with MLA (24 h) followed by Fluorescin-Aβ_1–42_ uptake (100 nM; 18 h) significantly decreased *Aβ*_***1–42***_-activated expression of *IL-1B* (Fig. 5b, left panel) and *TNFA* (Fig. 5b, right panel) in both lines suggesting an α7nAChR-dependent mechanism. Furthermore, Fluorescin-Aβ_1–42_ uptake increased expression of *IL-1β* and *TNF-α* in a concentration-dependent manner in *CHRFAM7A*-transfected cells but not in *EV*-transfected cells (Fig. 5c, left and right panels). The *CHRFAM7A*-dependent increase in IL-1β expression was validated by ICC (Fig. 5d) and immunoblotting (Fig. 5e). ELISA demonstrated that IL-1β is released from the cells and that the presence of CHRFAM7A causes a dose-dependent IL-1B release in response to Fluorescin-Aβ_1–42_ uptake (Fig. 5f).

## Discussion

Human-specific genes are emerging culprits in complex human diseases^46^. *CHRFAM7A*, a human specific fusion gene in high frequency in the population, has been implicated in a broad array of neuropsychiatric disorders, including schizophrenia, bipolar disorder, dementia with Lewy bodies, Pick disease, and AD; all are human-specific diseases affecting association cortices and higher cognitive function^25^. While CHRN7A has been a promising target for diseases affecting cognition, the effect observed in animal models failed to translate in human clinical trials suggesting a human-specific mechanism^13,14^, we developed a model system to study the modifying effect of CHRFAM7A which (i) has the human biological context, (ii) allows studies in specific cell types, (iii) is a renewable source, and iv) is amenable to scaling. Adapting this iPSC system for high-throughput screening can advance drug discovery in diseases such as AD and schizophrenia and addresses unmet medical needs. In this work, we present two iPSC lines, one with the rare homozygous ancestral haplotype with no *CHRFAM7A* and another harboring a single direct copy of *CHRFAM7A* on one allele with the ancestral haplotype on the other. The *CHRFAM7A* CNV harboring iPSC line contrasted to the 0 copy state provides a unique experimental system, where its effect can be studied in disease-relevant differentiated cells. While the two lines are from two different individuals, transfection of the *CHRFAM7A* into the 0 copy line showed similar results when the transfection efficiency is taken into account, suggesting that the results are independent of the genetic background. There is a need for genome-edited isogenic lines, such as UB068 with inserted *CHRFAM7A*, and UB052 with knocking out *CHRFAM7A* to address CHRFAM7A effect on α7nAChR function.

iPSC lines underwent stringent characterization and selected colonies were successfully differentiated into MGE progenitors. *CHRNA7* and *CHRFAM7A* expression increases with differentiation, and *CHRFAM7A* is preferentially induced when present in the cell line. Functional receptors demonstrated electrophysiological properties and response to pharmacological treatments with agonists, antagonists, and PAMs consistent with α7nAChR.

Interestingly, when *CHRFAM7A* was present either in the iPSC line UB052 or after transfection of HEK293 cells, electrophysiological properties of α7nAChR were different from the 0 copy line. PNU in UB068 cells (*CHRFAM7A* absent) progressively increased channel open probability of single α7nAChRs in a time-dependent manner^44^. On the other hand, in UB052, PNU-modulated currents ran down faster than in UB068. Comparative *n*Po analyses of currents from both cell types showed clear difference in kinetic properties whether CHRFAM7A was expressed or absent. When *CHRNA7* and *CHRFAM7A* cDNA in HEK 293 cells were expressed in 4:1 and 1:4 proportions, PNU modulated currents differentially. Qualitatively, the single-channel current clusters appeared similar in both cases; however, *n*Po analyses of both currents suggest that desensitization of PNU-modulated α7nAChR currents increased as a function of *CHRFAM7A* dosage. These altered electrophysiological properties could suggest that, based on their genotype, individuals may respond differentially to PAMs and perhaps to agonists and antagonists as well. Thus, during drug development by targeting α7nAChR for indications such as cognition or negative symptoms of schizophrenia, taking into account these pharmacogenetic correlations may prove more powerful.

*CHRFAM7A* modified Aβ_1–42_ uptake mediated through the α7nAChR^47–49,23^, demonstrating a dose response to Aβ_1–42_ concentration and modulation of uptake by α7nAChR agonist, antagonists, and PAMs. Comparison of the *CHRFAM7A* null (0 copy) and heterozygous (1 copy) lines and transfection of *CHRFAM7A* into the null line demonstrated a regulatory effect of *CHRFAM7A* on Aβ_1–42_ uptake, which was concentration dependent. Linear uptake of Aβ_1–42_ was observed in the 0 line, while the presence of *CHRFAM7A* mitigated the dose response of Aβ_1–42_ uptake at higher concentrations, suggesting a protective effect beyond physiological concentrations. These observations align with the age-dependent penetrance of AD and the hypothesis of decreased clearance being the leading mechanism in sporadic AD^50^. We hypothesize that CHRFAM7A is protective in AD during the accumulation of Aβ_1–42_ by mitigating Aβ_1–42_ uptake and by activating neuronal IL-1β expression and release as cry for help. Since α7nAChR targeting clinical trials focused on synaptic modulation and cognitive benefit in the short term (12–14 weeks), its suggested disease-modifying effect has not been explored.

These results suggest a negative modulatory effect of *CHRFAM7A* on synaptic transmission (relevance in schizophrenia) and a modulatory effect on Aβ_1–42_ uptake (relevance in AD), consistent with the direction of the association signals in schizophrenia (increased *CHRFAM7A* as risk) and AD (loss of *CHRFAM7A* as risk). Since the CNV is frequent, lead optimization may identify more potent molecules when the screen has a model with *CHRFAM7A*. Pharmacogenetics needs to be implemented in the clinical trials as the presence of *CHRFAM7A* will likely have an impact on drug effect. Further studies are needed to evaluate whether there is a dosage effect of *CHRFAM7A* when present on both chromosomes, and even three copy individuals have been detected. Cell lines with the inverted copy of *CHRFAM7A* could answer the long lingering question whether the inverted copy is expressed and have a functional effect on the α7nAChR or if it behaves as a null allele. Isogenic lines can eliminate the genetic heterogeneity concern and are being developed. Using a genetically relevant model system and translating it into a genetically characterized population could result in markedly improved signal and is consistent with the efforts to deliver precision medicine.

## Supplementary information

Supplementary data
