# Supplementary material for: iPSC model of CHRFAM7A effect on α7 nicotinic acetylcholine receptor function in the human context
Source: Transl Psychiatry. 2019 Feb 1;9:59. doi: 10.1038/s41398-019-0375-z (PMC6358606; doi:10.1038/s41398-019-0375-z)
Supplement: Supplementary file 1 — Supplementary data [file 41398_2019_375_MOESM1_ESM.pdf]

METHODS

Genotyping by CHRFAM7A breakpoint sequence TaqMan assay. TaqMan Copy Number Assay was performed to validate the copy number calls of *CHRFAM7A.* Primers (forward primer: GTAATAGTGTAATACTGTAACTTTAAAATGTGTTACTTGT, reverse primer: AGCCGGGATGGTCTCGAT) and probe (TCCTGACTGTACACATAAAA) were supplied by Applied Biosystems. The duplex real-time PCR assays were performed using a FAM dye-labeled assay targeted to 15q13.3 and the VIC dye-labeled RNaseP (TaqMan copy number reference assay, part # 4403326) as a reference gene. Each sample was assayed in quadruplicate by using 10 ng DNA in each reaction. Real-time PCR was performed using the CFX384 Real-time PCR Detection System (Bio-Rad). Threshold cycle (Ct) values were determined for *CHRFAM7A* and compared with Ct values for RNase P. Relative quantity was determined by the DD Ct method.

iPSC Generation and Cell Culture

iPSC lines (UB068- zero copy and UB052-one copy direct) were generated from human skin biopsies in WNYSTEM (University at Buffalo) by episomal transformation. iPSCs were grown on irradiated mouse embryonic fibroblasts in DMEM/F12-Glutamax medium supplemented with 10% KnockOut Serum Replacement, 1% Non-essential Amino Acids (NEAA), and 0.1% 2-Mercaptoethanol (all *Thermo Fisher*). Cells were maintained at $37^{o}C/5\%$ CO_2 and subcultured every 4-6 days using Dispase (*Thermo Fisher*).

iPSC Characterization

Live staining: UB068 and UB052 colonies were stained with TRA-1-60 Alexa Fluor 488 conjugate kit (Life Technologies) according to the manufacturer's protocol. Live images were taken by EVOS microscope *(Life Technologies*) with 20x or 40x objectives.

Gene and protein expression: Expression of the pluripotency/self-renewal and the three germ layer markers at gene and protein levels was assessed by RT –qPCR and immunocytochemistry (Supplementary data, Methods; The primers (*IDT*) are listed in Supplementary data, Table 1, The primary antibodies are listed in Supplementary data, Table 2).

Array comparative genome hybridization (aCGH): aCGH between the iPSC colony and the original blood DNA sample from the same individual according to the manufacturer's protocol. The ADEM2 algorithm with a threshold of 6 was used to detect de novo events.

TaqMan hPSC Scorecard Assay: Pluripotency of the iPSC was evaluated by the TaqMan hPSC Scorecard Assay *(Life Technologies)* according to the manufacturer's protocol. (32) (33).

Neuronal Differentiation and Transfection

Neuronal differentiation of iPSC towards Medial Ganglionic Progenitors (MGE) and neurons was carried out using the protocol based on Liu et al (16) with modifications. Undifferentiated UB068 and UB052 cells were detached at day 0 and continued to float in T-25 flasks with Essential 6 medium (E6 medium) (*Life Technologies*) on the slow rotary shaker. iPSC formed Embryoid Bodies (EB) at day 2. The medium was replaced with neural induction medium (NIM) [DMEM/F12+Glutamax, N2 (*Life Technologies*), NEAA (*Gibco*), Heparin (*Stem Cell Technonlogies*) and pen/strep] on day 4. EB were attached in 6-well plates on day 7, and by day 10 neural rosettes were present, indicating the development of primitive neuroepithelia. Ventralization of primitive neuroepithelia was started at day 10 by adding Purmorphamine, Pur (1.5 μM). On day 16, neural tube-like rosettes were detached and transferred into T-25 Flasks in

NIM with 2% B27 (*Life Technologies*) to form neurospheres. The cultures were fed every other day. On day 25, the neural progenitor cells, NPCs, were dissociated with Accutase (*Stem Cell Technonlogies*) and plated onto 6-well plates for characterization, expansion, and further differentiation to GABA interneurons and BFCN.

Quantitative Polymerase Chain Reaction (qPCR)

Total RNA was isolated from cell cultures at different stages of neuronal differentiation (D0 pluripotent stem cells, D25- neural progenitor cells, D40 – neurons) using Trizol (*Invitrogen*) according to manufacturer's protocol. cDNA was synthesized from 500ng of total RNA using ImProm-II reverse transcriptase (*Promega*) and oligo (DT) (*Promega*). The reaction was carried out at 42°C for one hour. For quantitative gene expression, standard RT-qPCR was performed using the primers (*IDT*) listed in Supplementary data, Table 1. qPCR was performed using the SYBR green master mix (*Biotool*) and run on Bio-Rad CFX Connect cycler *(Bio-Rad)*. Samples were assayed with 3 technical replicates, and data was analyzed using the $\Delta\Delta C_T$ method and normalized to GAPDH expression. Data are presented as the average of the triplicates ± standard error of the mean.

Immunocytochemistry and Confocal Microscopy

Cells plated on glass coverslips or 8-well glass chambers (*ThermoFisher*) were fixed with 4% paraformaldehyde (*Mallinckrodt Baker*) for 15 minutes, permeabilized with 0.1% Triton X100 (*Mallinckrodt Baker)* for 10 minutes, and blocked with blocking buffer (5% BSA in PBS) for 1 hour at room temperature (RT). Cells were incubated overnight at 4°C with primary antibodies (Supplementary data, Table 2). On the next day, cells were incubated for 1h at RT with secondary antibodies. Both primary and secondary antibodies were diluted in blocking

buffer. Slides were mounted with a Prolong ® Gold antifade reagent with DAPI (*Life Technologies*), and confocal images were captured by using LSM510 Meta microscope (40 x objective). Images were acquired using ZEN black software.

Electrophysiology

Most electrophysiological experiments were done ~24 hrs post-transfection. Single-channel currents were recorded in the cell-attached patch configuration (23° C). The bath solution was (in mM) 142 KCl, 5.4 NaCl, 1.8 $CaCl_2$, 1.7 $MgCl_2$, 10 HEPES/KOH (pH 7.4). Because of the high extracellular [K^+], the membrane potential V_m was ~0 mV. In most cases (except in action current recordings), we routinely added 100 nM Tetrodotoxin (TTX) and 10 mM tetraethylammonium (TEA) to block Na_v and K_v currents.

Patch pipettes were fabricated from borosilicate glass, coated with sylgard (Dow Corning, Midland, MI) and fire polished to a resistance of ~10 MΩ when filled with pipette solution (Dulbecco's phosphate-buffered saline PBS) (in mM): 137 NaCl, 0.9 $CaCl_2$, 2.7 KCl, 1.5 KH_2PO_4, 0.5 $MgCl_2$, and 8.1 Na_2HPO_4 (pH 7.3/ NaOH). Single channel currents were recorded using a PC505 amplifier (Warner instruments, Hamden, CT), low-pass filtered at 20 kHz and digitized at a sampling frequency of 50 kHz using a NI data acquisition board (SCB-68, National instruments, Austin, TX).

For ligand-activation/modulation experiments, Acetylcholine,, MLA, Memantine (all from *Millipore Sigma*), and PNU 120569 *(Tocris*) were added to the pipette solution in desired concentrations from a stock solution. The membrane potential (V_m) was -100 mV when low [agonist] were used and +100 mV when high [agonist] were used to relieve channel block.

Kinetic analyses of single channel currents were performed by using QuB {Milescu, 2003 #172} . Single channel currents were idealized by segmental k-means algorithm (SKM). nPo was

estimated by dividing the cumulative open probability by the number of channels in the patch (maximum number of overlaps of open current levels in the data) as follows:

$$Po = \frac{\sum nPo}{\mathrm{n}}$$

Table S1. Primer Sequences used for RT-qPCR

Gene	Forward Sequence	Reverse Sequence	Reference
NANOG	TGCAAATGTCTTCTGCTGAGAT	GTTCAGGATGTTGGAGAGTTC	Sundberg et al., 2011
OCT-4	CGTGAAGCTGGAGAAGGAGAAGCTG	AAGGGCCGCAGCTTACACATGTTC	Sundberg et al., 2011
SOX2	AATAGACCTAAGCCATGTGACATCC	GCAAACTTCCTGCAAAGCTC	Sundberg et al., 2011
PAX6	ACCACACCGGTTTCCTCCTTCACA	TTGCCATGGTGAAGCTGGGCAT	Imaizumi et al., 2015
NKX2.1	CGCATCCAATCTCAAGGAAT	CAGAGTGTGCCCAGAGTGAA	Liu et al., 2013
LHX8	CCAAAACCAGCAAAAAGAGC	TGGCGTGCTCTACAATTCTG	Liu et al., 2013
LHX6	ACAGATCTACGCCAGCGACT	CATGGTGTCGTAGTGGATGC	Liu et al., 2013
FOXG1	AGAAGAACGGCAAGTACGAGA	TGTTGAGGGACAGATTGTGGC	Bissonnette et al., 2013
MAP2	AATAGACCTAAGCCATGTGACATCC	AGAACCAACTTTAGCTTGGGCC	Sundberg et al., 2011
ChAT	TGTCTGAGGAGCAGTTCAGG	GGCCTTGTAGCTGAGTACACC	Crompton et al., 2013
GAD	GTCGAGGACTCTGGACAGTA	GGAAGCAGATCTCTAGCAAA	*Life Technologies*
TH	AGCTCCTGAGCTTGTCCT TG	TGTCCACGCTGTACTGGTTC	Hermann et al., 2004
HB9	GCACCAGTTCAAGCTCAAC	GCTGCGTTTCCATTTCATCC	Liu et al., 2013
TUBB3	TGTACTCCTTCCTGCTGGACTT	CCCCAACTCTCACTATGTGGAT	Fan et al., 2014
GATA4	TCATCTCACTACGGGCACAG	GGGAAGAGGGAAGATTACGC	Fan et al., 2014
SOX17	CTTTCATGGTGTGGGCTAAGG	GTACTTGTAGTTGGGGTGGTCCT	Fan et al., 2014
FOXA2	GAAGATGGAAGGGCACGA	CACGTACGACGACATGTTCA	Fan et al., 2014
MEOX1	AGAGTTTGCCCATCATAACTACCT	GCTCAGTCCTTAGTCATTTTTCCTC	Fan et al., 2014
IL1B	GGAGAATGACCTGAGCACCT	GGAGGTGGAGAGCTTTCAGT	
TNFA	GTCAACCTCCTCTCTGCCAT	CCAAAGTAGACCTGCCCAGA	
NFKB	CTACGACCTGAATGCTGTGC	CTGCCAGAGTTTCGGTTCAC	
CAS1	ATGGCCGACAAG GTCCTG A	TTTAATGTCCTGGGAAGAGGTAGA	
CAS8	CATCCAGTCACTTTGCCAGA	GCATCTGTTTCCCCATGTTT	
NLRP3	ATGAAGATGGCAAGCACCCG	CTACCAAGAAGGCTCAAGACGAC	
GAPDH	GTTCGACAGTCAGCCGCATC	GGAATTTGCCATGGGTGGA	Sundberg et al., 2011

Table S2. Anti-human antibodies used for immunocytochemistry

Antibody	Species	Supplier	Dilution
α - Nanog	Mouse	Cell Signaling Technology	1:200
α – Oct-4	Rabbitt	Cell Signaling Technology	1:400
α – Sox2	Rabbit	Cell Signaling Technology	1:400
α – SSEA-4	Mouse	DSHB	1:50
α - MAP2	Mouse	R&D Systems	1:250
α - NKX2.1	Rabbit	Abcam	1:250
α – GABA	Rabbit	Millipore-Sigma	1: 1000
α – ChAT	Rabbit	Millipore-Sigma	1:1000
α – α7nAChR	Mouse	Millipore-Sigma	1:500
α – IL-1β	Mouse	LSBio	1:200
α – βIII-tubulin	Rabbit	Cell Signaling Technology	1:200
α – αSMA	Mouse	Santa Cruz Biotechnology	1:200
α – αAFP	Mouse	R&D Systems	1:250

Figure 1. Characterization of new iPSC lines

A - Representative image of microarray analysis. Comparison of genomic DNA from an iPSC colony (UB068) to original blood DNA from the same individual. No chromosomal abnormalities were identified. The experimental procedures on the Agilent 244 k array are performed according to the manufacturers' instructions. Normalized $\log_2$-ratio data are generated by the manufacturer's microarray scanner and quantification software (CGH analytics, Agilent) and segmentation is performed with the ADM-2 algorithm implemented in the Cytogenomics software.

B - TaqMan copy number variation assay shows relative amount of CHRFAM7A in UB068 (0 copy) and UB052 (1 copy) lines.

C - Representative live images of iPSC undergoing non-directed three germ layer differentiation (D3 – EB formation; D10, D15 – generation of the three germ layers at D10 and D15 of non-directed differentiation).

D - hPSC Scorecard™ Data Analysis Report showing fold change in expression of the individual gene relative to undifferentiated reference set.

Figure 2. Neuronal differentiation of iPSC

A – RT-qPCR analysis of gene expression patterns during neuronal differentiation of iPSC. Expression levels of *NANOG* (pluripotency marker) *NKX2.1*, *LHX6*, and *LHX8* (MGE markers), *PAX6 (*dorsal forebrain marker*) SOX2,* and *MAP-2* (pan neuronal markers), *ChAT*, *GAD*, *TH*, and *HB9* (BFCN, GABA, dopaminergic, and motor neuron markers, respectively) were detected throughout neuronal differentiation of UB068 and UB052 cells.

B – Representative confocal images of BFCN (ChAT staining) and GABA interneurons (GABA staining) at day 40 of neuronal differentiation.

C - Whole cell patch clamp confirming the presence of functional neurons. Spontaneous action current activity recorded from UB068-derived neurons in cell-attached patch-clamp experiments (upper panel). Representative traces of whole cell current recording from the UB068 cell line in response to voltage steps from -80mV to +30mV in increments of 10mV (middle panel). Current-Voltage relationship curve (n=8). Currents normalized to +30mV (lower panel).

D - Functional expression of neuronal α7 nAChRs studied by recording cell-attached single channel currents from UB068-derived neurons at 40 of differentiation. The effect of increasing concentrations of MLA on α7 nAChR currents: MLA blocks α7 nAChRs in a dose-dependent manner and the block could be relieved by reversing the membrane voltage.

Figure 3. Overexpression of CHRFAM7A-mCherry in UB068 neurons at 72 h after transfection. Expression level of CHRNA7 is not affected by transfection with pcDNA3.3-mCherry or CHRFAM7A-mCherry. Inset: FITC-$Ab_{1\text{-}42}$ uptake (green) in transfected UB068-derived neurons (red).

Figure 4. Fluorescin-$A\beta_{1–42}$ uptake in pcDNA3.1-CHRFAM7A-mCherry (CHRFAM7A) or pcDNA3.3-mCherry control (EV) transfected iPSC-derived neurons. Approximately ~10^4 cells were analyzed by flow cytometry and representative histograms of total gated events for median samples are presented. Gated on a uniform, normally distributed FSC-A/SSC-A population with doublet discrimination through secondary FSC-A/FSC-H analysis.

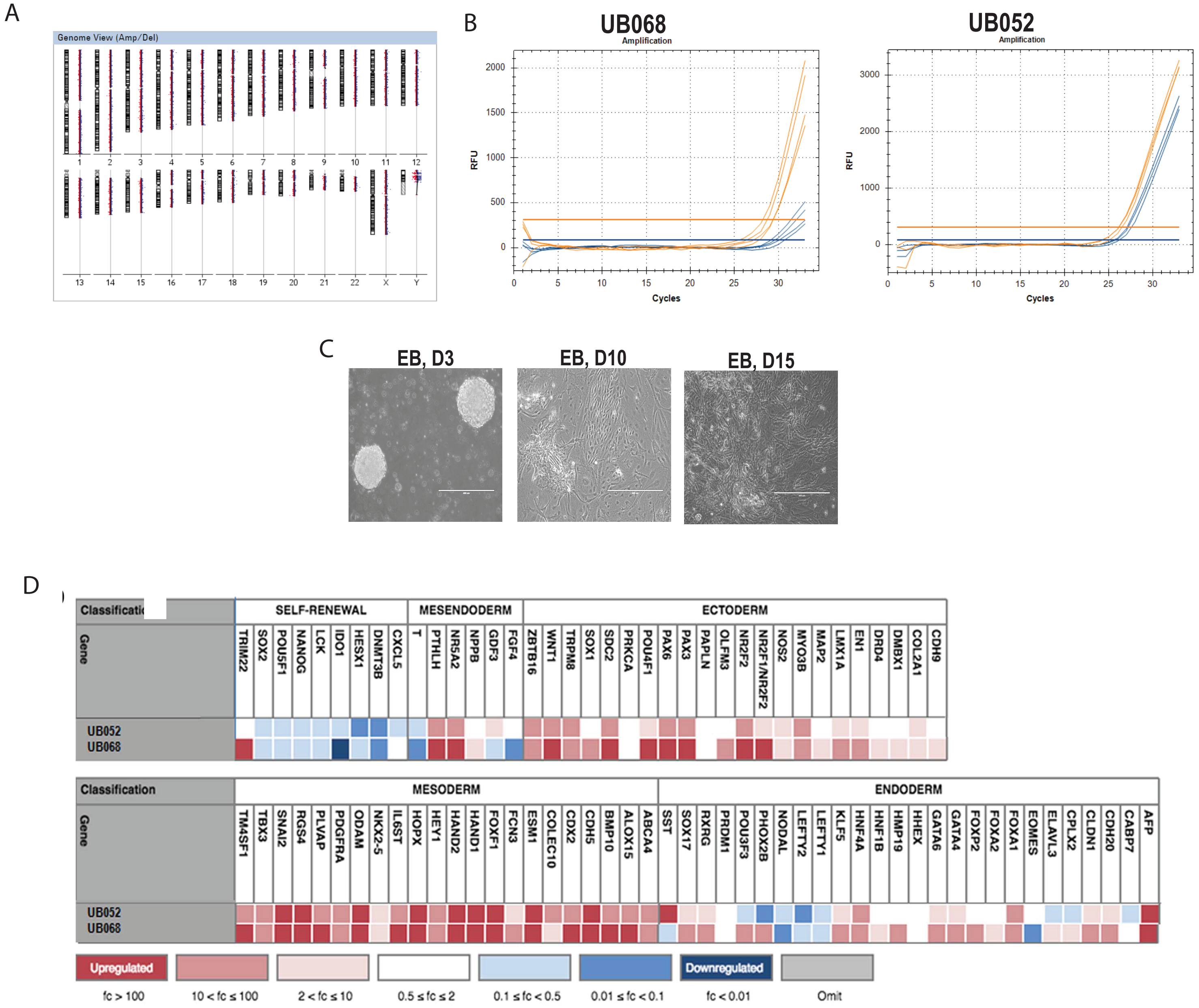

Supplementary Figure 1

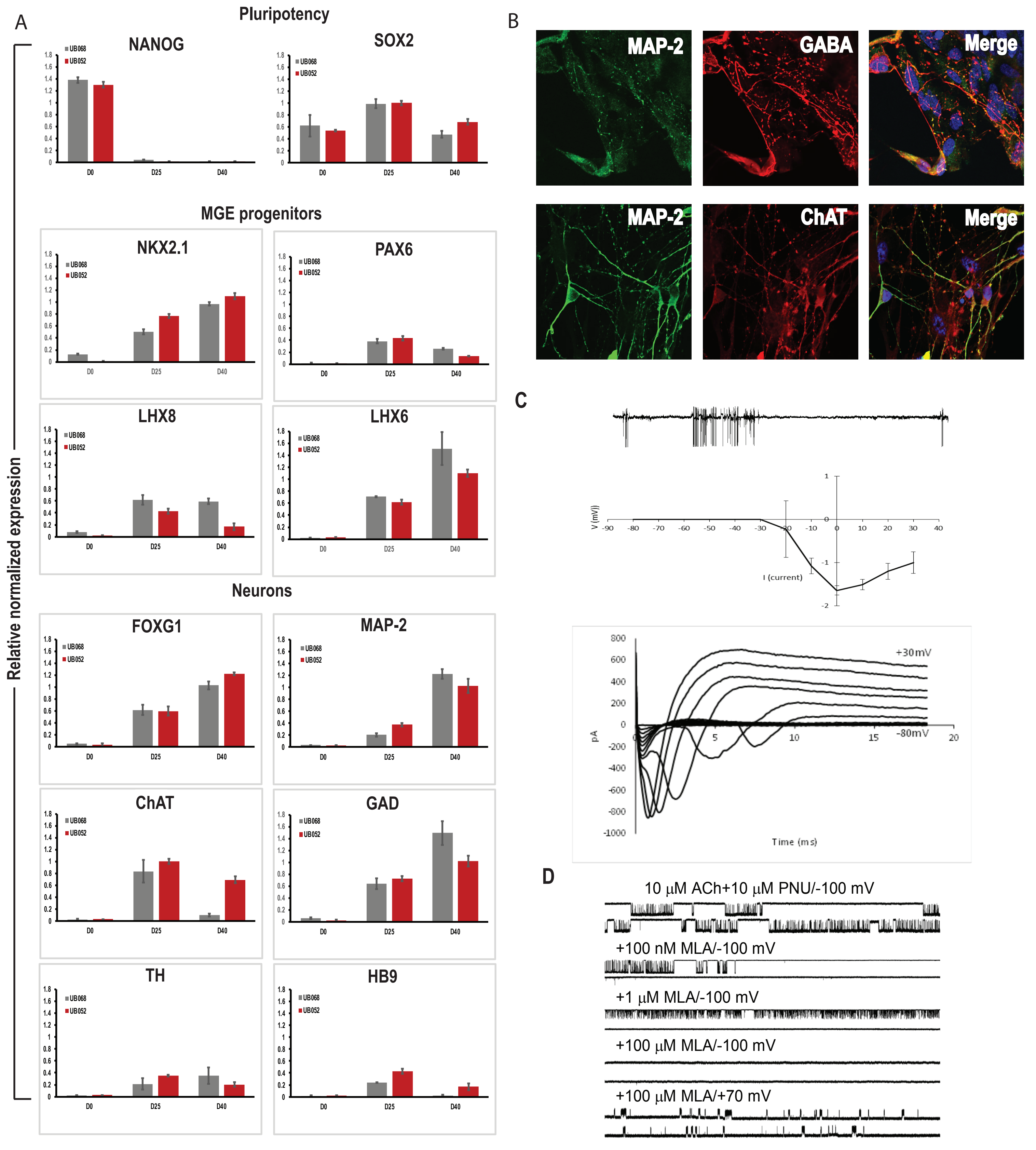

Supplementary Figure 2

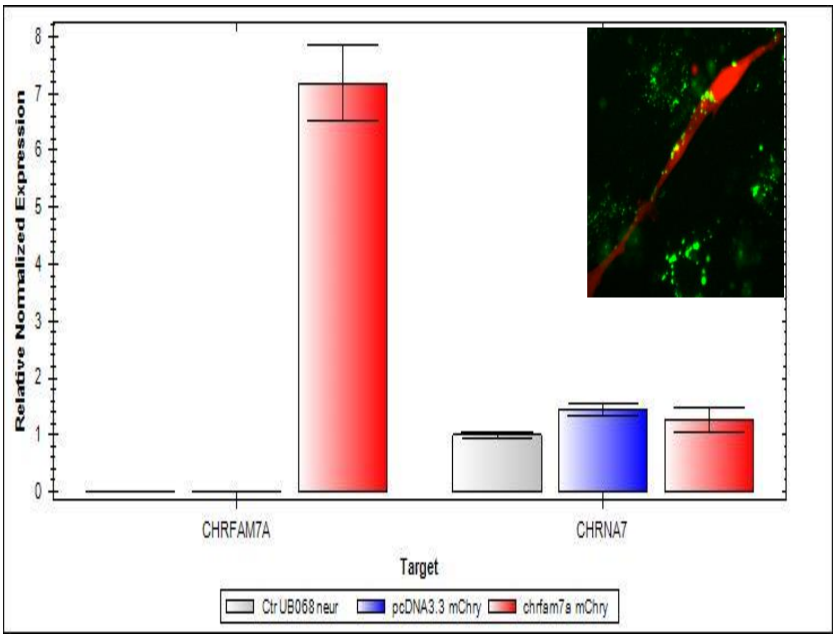

Supplementary Figure 3

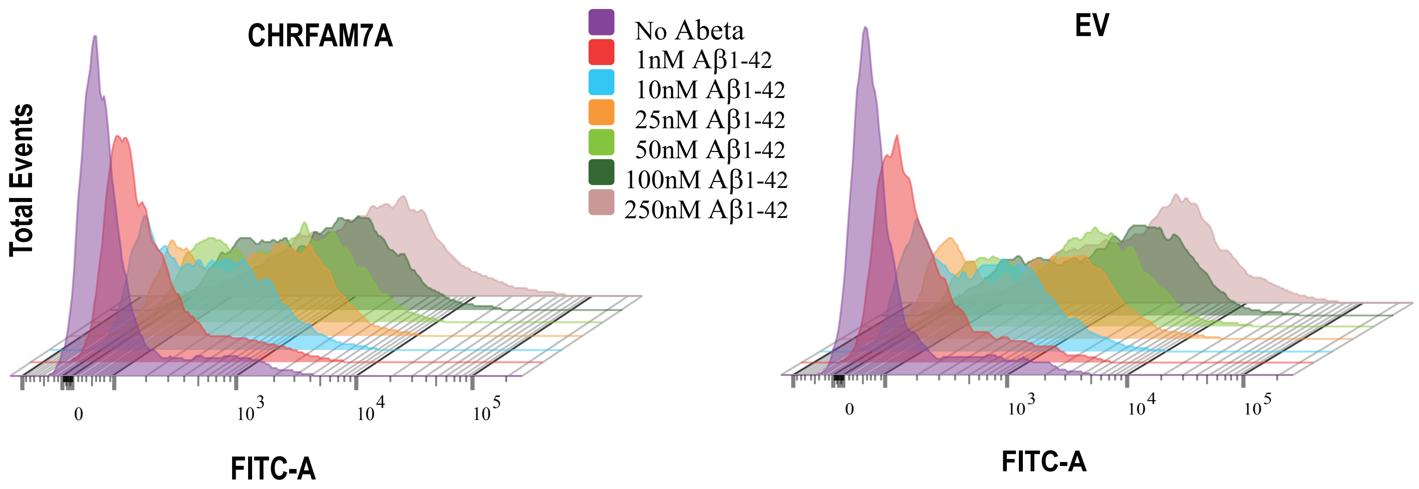
CHRFAM7A
EV
Total Events
FITC-A
FITC-A
0
10^3
10^4
10^5
No Abeta
1nM Aβ1-42
10nM Aβ1-42
25nM Aβ1-42
50nM Aβ1-42
100nM Aβ1-42
250nM Aβ1-42
